# Dipeptide tyrosyl-leucine exhibits antidepressant-like activity in mice

**DOI:** 10.1038/s41598-020-59039-7

**Published:** 2020-02-10

**Authors:** Takafumi Mizushige, Tomoki Uchida, Kousaku Ohinata

**Affiliations:** 1 Division of Food Science and Biotechnology, Graduate School of Agriculture, Kyoto University, Gokasho Uji, Kyoto 611-0011 Japan; 2 Research Unit for Physiological Chemistry, C-PIER, Kyoto University, Kyoto, 606-8501 Japan; 3 Department of Applied Biological Chemistry, Faculty of Agriculture, Utsunomiya University, 350 Minemachi, Utsunomiya, Tochigi 321-8505 Japan

**Keywords:** Peptides, Stress and resilience

## Abstract

Depression is a worldwide health problem. In the present study, we found that a dipeptide, tyrosyl leucine (Tyr-Leu, YL), administered orally, intracerebroventricularly, or intraperitoneally exhibited a potent antidepressant-like activity in the forced swim and tail suspension tests in naïve mice. YL increased the amount of cells expressing c-Fos, a marker for neuronal activity, in the dentate gyrus of the hippocampus. YL increased bromo-2′-deoxyuridine-positive cells and doublecortin expression in the dentate gyrus of the hippocampus, suggesting that YL enhanced the proliferation of hippocampal progenitor cells *in vivo* and *in vitro*. YL did not affect hippocampal mRNA and protein expression of BDNF, which is a regulatory factor of both neurogenesis and depression-like behavior. Intriguingly, YL suppressed activation of the hypothalamo-pituitary-adrenal axis by forced swim stress. Moreover, other aromatic amino acid-leucines, Phe-Leu and Trp-Leu, also exhibited antidepressant-like activities, suggesting that the structure of aromatic amino acid-leucine may be important for antidepressant activity. In addition, bovine milk casein-derived peptide, Tyr-Leu-Gly (YLG), an anxiolytic peptide, exhibited an antidepressant-like activity. Our findings demonstrate that YL exhibits an antidepressant-like effect, moderates the stress response, and induces hippocampal neuronal proliferation through a signal pathway independent of BDNF.

## Introduction

A number of bioactive peptides have been found in enzymatic digests of various food proteins, and some of these peptides are known to act on the nervous system. We also reported that bioactive peptides derived from food proteins exhibit anxiolytic-like effects^1–7^, suggesting interactions between food components and the nervous system. Clarification of these interactions is challenging, since the food components, including food protein digests, consist of numerous molecular species. On the other hand, we have found novel bioactive peptides based on the structure-activity relationships of dipeptides. Thus, the discovery of dipeptides with potent activities and the their structure-activity relationships are very important and powerful clues to find novel peptides. Indeed, we initially found that a dipeptide, tyrosyl leucine (Tyr-Leu, YL), had a potent anxiolytic-like effect, comparable to that of diazepam, one of the general anxiolytics, in mice^1^. Thereafter, based on the structure rule required for the anxiolytic activity of YL, novel food-derived peptides have been rapidly been identified. Thus, we focused on dipeptides.

Major depressive disorder, one of the most common psychiatric diseases, is characterized by dysregulation of emotion and mood as well as abnormalities of cognitive function, sleep, appetite, and metabolism. It is known to be a leading cause of disability worldwide^8,9^. Anxiolytic molecules often exhibit antidepressant-like effects. Thus, in the current study, we investigated whether YL exhibits antidepressant-like activities using two behavioral tests commonly used to screen antidepressants, the forced swim and tail suspension tests.

Some antidepressant drugs were reported to promote hippocampal neurogenesis. It is known that unpredictable mild stress decreases the neural activity of hippocampal cells and the proliferation of hippocampal progenitor cells, and that these activities can be increased by the administration of antidepressants^10–12^. We then examined the hippocampal c-Fos protein-positive cells, known as a general neural activity marker^13–15^, and the effects of the proliferation of progenitor cells *in vivo* and *in vitro*. Brain-derived neurotrophic factor (BDNF) is known to induce the proliferation of hippocampal progenitor cells and has an antidepressant-like effect^16–18^. We also examined the expression of BDNF after administration of YL.

The hypothalamo-pituitary-adrenal (HPA) axis is neuroendocrine response to stressors and releases corticotropin-releasing hormone (CRH) and corticosterone^19^. Excess stress and impairment of the HPA axis system induce depressive behaviour and inhibit proliferation of neural stem cell. It has been reported that some antidepressants improved this abnormal stress response^20,21^. Thus, we investigated whether YL inhibited stress-induced HPA axis activation by evaluating hypothalamic CRH expression and plasma corticosterone levels.

Mediators of YL-induced antidepressant-like effects were also investigated. We previously revealed that a novel signaling pathway with serotonin 5-HT_1A_ receptor activation followed by the activation of dopamine D_1_ and GABA_A_ receptors mediate antidepressant-like and anxiolytic-like activities^1,6^. We also found that this signaling pathway mediates the anxiolytic effect of YL. Thus, we tested whether the antidepressant-like activity of YL is also mediated by this common pathway.

In addition, we investigated the structure rules required for the antidepressant-like effect using YL analogues. We also tested the antidepressant-like effect of tyrosyl leucyl glycine (Tyr-Leu-Gly, YLG), a tripeptide found to be a potent anxiolytic peptide that is released after pepsin-pancreatic digestion, mimicking the gastrointestinal enzymatic conditions of α_S1_-casein, a major bovine milk protein^22^.

In this study, we investigated the effect of YL on antidepressant-like behaviour and its mechanisms. Neuronal cell proliferation in the hippocampus *in vivo* and *in vitro*, BDNF and HPA-related factors, and mediators of YL-induced antidepressant-like effect were investigated biochemically and pharmacologically. The structure-activity relationships of antidepressant-like activity were also examined.

## Results

**Figure 1:**
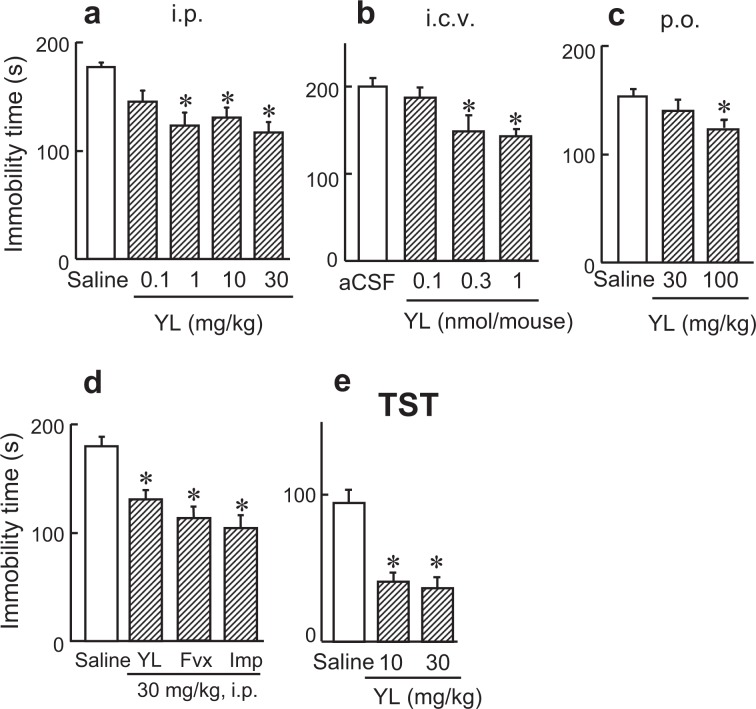
YL exhibits an antidepressant-like effect in mice. (**a**–**c**) Antidepressant-like effect of YL. YL ((**a**) 0.1–30 mg/kg, i.p.; (**b**) 0.1–1.0 nmol/mouse, i.c.v.; (**c**) 30–100 mg/kg, p.o.) was administered to mice, and the immobility time during a 4-min forced swim test (FST) session was measured 30 min after administration (n = 11; n = 8–9; n = 18, respectively). (**d**) Antidepressant-like activity of YL was comparable to those of already-known antidepressants. Immobility time during a 4-min FST session was measured after the administration of YL, fluvoxamine, and imipramine (30 mg/kg, i.p.) in mice (n = 7–8). (**e**) Antidepressant-like effect of YL (10–30 mg/kg, i.p.) in the tail suspension test (TST) (n = 6).

To investigate antidepressant-like effects, we performed the forced swim test (FST) and tail suspension test (TST), used for screening antidepressants, in mice. YL, a simple dipeptide composed of l-tyrosine and l-leucine, decreased immobility time after intraperitoneal (F(4, 50) = 7.256, *p* < 0.05, 1–30 mg/kg) and intracerebroventricular (F(3, 32) = 6.457, *p* < 0.05, 0.3–1 nmol/mouse) administrations in the FST (Fig. 1a,b). These results suggest that YL exhibits an antidepressant-like effect in the FST. YL was also orally active (F(2, 35) = 3.196, *p* < 0.05, Fig. 1c). The YL-induced antidepressant-like activity was comparable to that of conventional antidepressants such as imipramine and fluvoxamine (F(3, 36) = 11.163, *p* < 0.05, Fig. 1d). In the TST, another behavioral test, YL also significantly decreased immobility time (F(2, 24) = 17.804, *p* < 0.05, Fig. 1e), suggesting that it mimics antidepressants in both the FST and TST. YL exhibited an antidepressant effect in two mouse strains, C57BL/6 N and ddY, in the FST (data not shown). Thus, we demonstrated that YL exhibits an antidepressant effect in two different paradigms in two different strains of mice. YL did not affect locomotor activity in the elevated plus maze and open field tests^1^.

**Figure 2:**
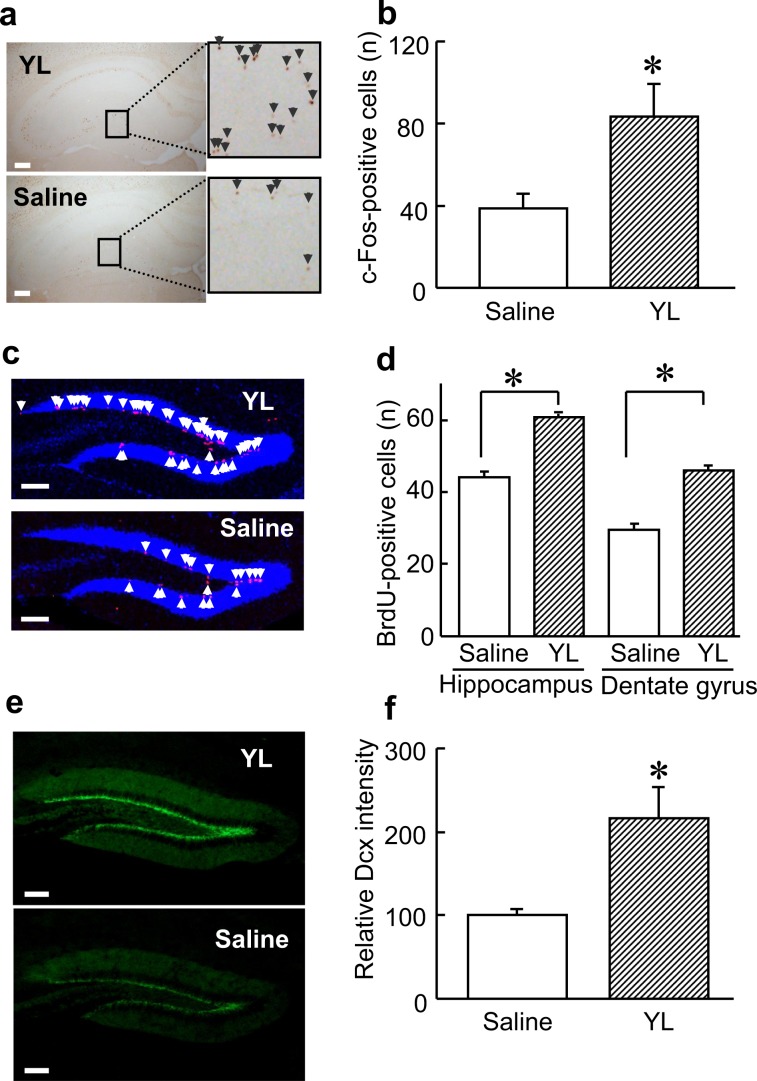
YL activates hippocampal cells and exhibits cell proliferation in the hippocampal dentate gyrus *in vivo*. (**a**,**b**) Immunostaining of the c-Fos protein. Hippocampal neuronal activity was evaluated by counting c-Fos-positive cells (n = 4). Scale bar, 100 μm. Values are expressed as the mean ± S.E.M. *P < 0.05 compared with each group by nonparametric Mann–Whitney U test. (**c**,**d**) Co-immunofluorescence staining of BrdU (red) and DAPI (blue). Hippocampal neuronal cell proliferation was evaluated by counting BrdU-positive cells in the whole hippocampus and dentate gyrus (n = 4). Values are expressed as the mean ± S.E.M. *P < 0.05 compared with each group by nonparametric Mann–Whitney U test. Scale bar, 100 μm. (**e**,**f**) Immunofluorescence staining of Dcx (green). Hippocampal neural maturation was evaluated by immunofluorescence intensity using Image J software (n = 4). Scale bar, 100 μm. Values are expressed as the mean ± S.E.M. *P < 0.05 compared with each group by nonparametric Mann–Whitney U test.

It was reported that antidepressants enhance adult proliferation of progenitor cells in the dentate gyrus of the hippocampus in rodents^11^. Indeed, YL administration increased the amount of cells expressing the c-Fos protein, a marker of neuronal activity, in the dentate gyrus of the hippocampus (*p* < 0.05, Mann-Whitney U test, Fig. 2a,b). We then investigated whether YL stimulates neural cell proliferation in the hippocampus. YL increased bromodeoxyuridine (BrdU)-positive puncta in the mouse hippocampus (*p* < 0.05, Mann-Whitney U test, Fig. 2c,d), and this increase was prolific in the dentate gyrus, where the proliferation of hippocampal progenitor cells was reported^11^. YL also increased the immunostaining intensity of doublecortin (DCX), a marker of newly differentiated neurons (*p* < 0.05, Mann-Whitney U test, Fig. 2e,f), in the hippocampus, where BrdU-positive cells were increased. These results suggest that YL promotes hippocampal cell proliferation and neuronal maturation *in vivo*.

**Figure 3:**
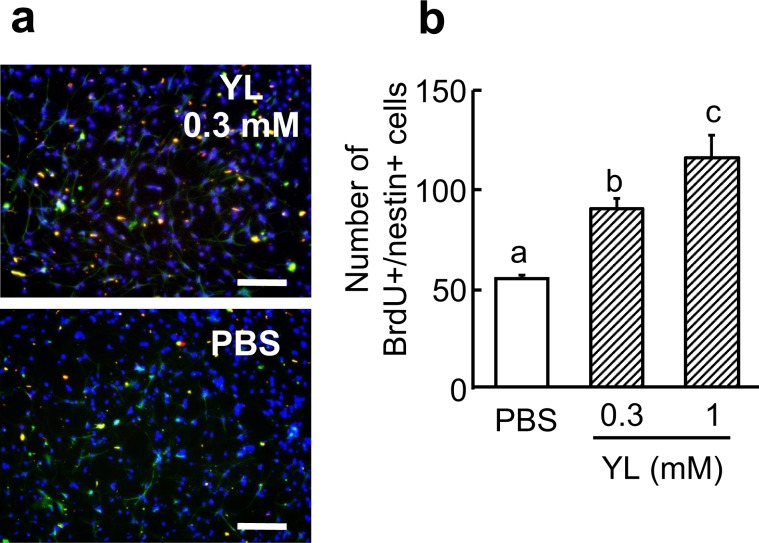
YL promotes proliferation of hippocampal neural stem cell *in vitro*. (**a**,**b**) Co-immunofluorescence staining of BrdU (red), nestin (green), and DAPI (blue). Hippocampal neural stem cell proliferation was evaluated by counting BrdU and nestin-positive cells in the culture (n = 4). Values are expressed as the mean ± S.E.M. Different characters represent significant differences (P < 0.05) among groups by the nonparametric Mann–Whitney U test. Scale bar, 100 μm.

Next, we examined the proliferation activity of hippocampal neural stem cells on YL *in vitro*. YL increased the number of BrdU and nestin-double positive cells in neural stem cell culture (Fig. 3a,b). Thus, YL may directly promote hippocampal cell proliferation.

**Figure 4:**
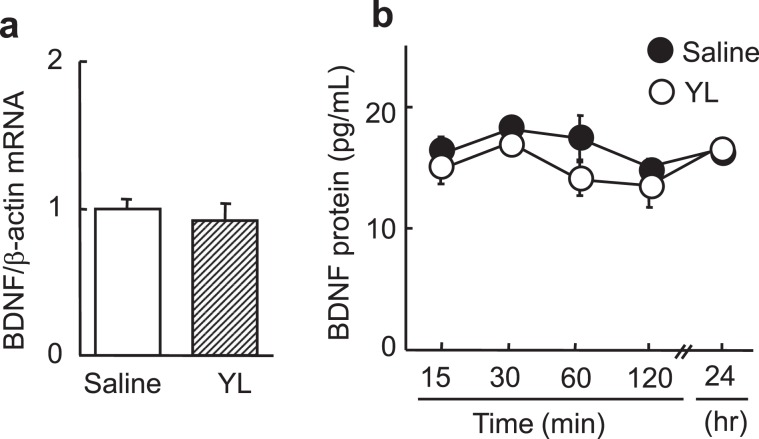
YL does not activate BDNF systems. (**a**) Hippocampal BDNF mRNA expression normalized to β-actin 30 min after i.p. administration of YL (30 mg/kg, n = 7). (**b**) Changes in hippocampal BDNF protein levels after YL administration (30 mg/kg, i.p., n = 7). Values are expressed as the mean ± S.E.M. *P < 0.05 compared with each group by ANOVA followed by Tukey-Kramer’s test.

We also tested involvement of BDNF in the antidepressant-like effect of YL. There were no changes in BDNF mRNA expression or protein levels after YL administration (Fig. 4a,b), suggesting that the effects on neuronal cell proliferation and the antidepressant-like effect were independent of the BDNF system.

**Figure 5:**
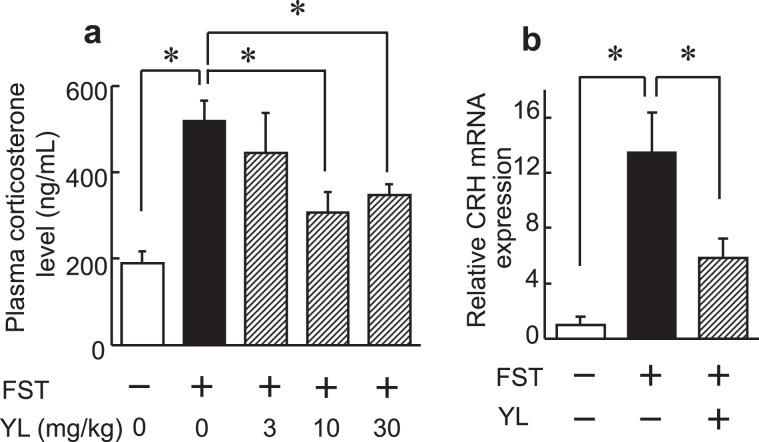
(**a**) Plasma corticosterone levels with and without the FST 30 min after i.p. administration of YL (3–30 mg/kg, n = 11–12). (**b**) Hypothalamic CRH mRNA expression normalized to β-actin with and without the FST after i.p. administration of YL (30 mg/kg, n = 4). Values are expressed as the mean ± S.E.M. *P < 0.05 compared with each group by ANOVA followed by Tukey-Kramer’s (**a**) or nonparametric Mann–Whitney U (**b**) tests.

Stress response was related to the regulation of depressive behavior. Excessive stress increases depression-like behavior. We then investigated whether YL suppresses the stress response induced by forced swim stress. Forced swim stress for 6 min increased the serum corticosterone concentration (F(4, 35) = 9.878, *p* < 0.05) and hypothalamic CRH mRNA expression (*p* < 0.05, Mann-Whitney U test, Fig. 5a,b). YL administration decreased the serum corticosterone concentration and hypothalamic CRH mRNA expression in the stressed mice (Fig. 5a,b). These results suggested that YL suppressed the stress response of the HPA axis.

**Figure 6:**
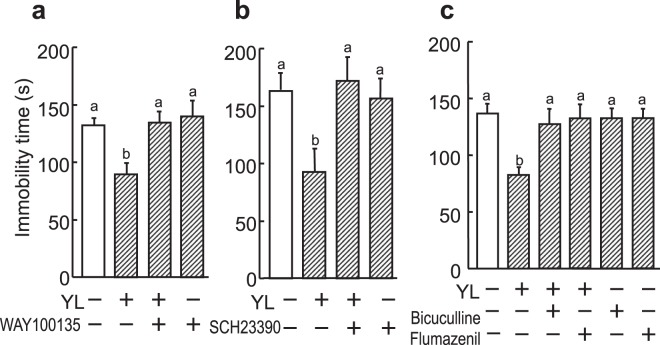
YL activates the 5-HT_1A_, dopamine D_1_, and GABA_A_ systems. Effect of serotonin 5-HT_1A_, dopamine D_1_, and GABA_A_ antagonist on YL-induced antidepressant-like activity. WAY100135 dihydrochloride, a serotonin 5-HT_1A_ receptor antagonist (10 mg/kg), R(+)-SCH-23390 hydrochloride, a dopamine D_1_ receptor antagonist (30 mg/kg), or (−)-bicuculline (5 mg/kg) or flumazenil, antagonists of GABA- and benzodiazepine-binding sites of GABA_A_ receptors, respectively (1 mg/kg), was co-administered once intraperitoneally with the peptide 30 min before FST. Values are expressed as the mean ± S.E.M. (n = 9–10). Different characters represent significant differences (P < 0.05) among each group by ANOVA followed by Tukey-Kramer’s test.

To investigate whether the YL-induced antidepressant-like effect is mediated via the activation of serotonin 5-HT_1A_, dopamine D_1_, and GABA_A_ receptors, which are well known to be involved in emotional behavior, we used antagonists specific for these receptors. The antidepressant-like activity of YL was blocked by WAY100135 (F(3, 22) = 6.218, *p* < 0.05, Fig. 6a), SCH23390 (F(3, 22) = 5.489, *p* < 0.05, Fig. 6b), bicuculline, or flumazenil (F(5, 54) = 4.438, *p* < 0.05, Fig. 6c), antagonists of 5-HT_1A_, dopamine D_1_, and GABA- and benzodiazepine-binding sites of GABA_A_ receptors. YL had no affinities for these receptors^1^, suggesting that these neurotransmitters may be released in association with the YL-induced antidepressant-like effect.

**Figure 7:**
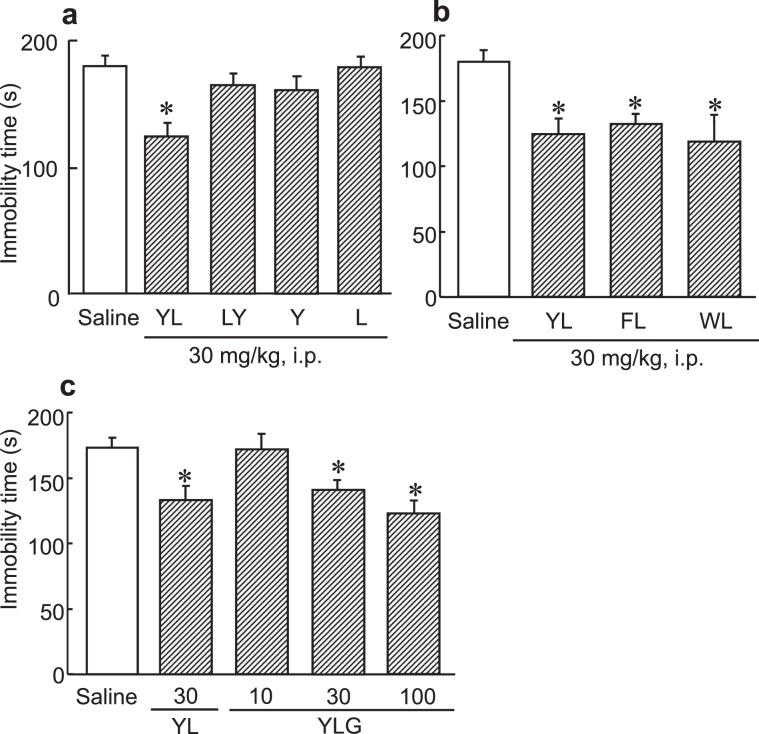
Antidepressant-like activity of the structure-activity relationship of YL. (**a**) Immobility time during a 4-min FST session when YL, LY, Y, and L (30 mg/kg body weight) were administered intraperitoneally to mice (n = 7–8). Value are expressed as the means ± S.E.M. Asterisks show significant differences between the YL-administered group and the vehicle-administered group. *P < 0.05 as determined by ANOVA. (**b**) Structure-activity relationship of the antidepressant-like effect of YL in the FST. YL, FL, and WL (30 mg/kg, i.p.) were administered to mice and immobility time during a 4-min FST session was measured (n = 7–8). (**c**) YLG (10–100 mg/kg, i.p.) and YL (30 mg/kg, i.p.) were administered to mice, and immobility time during a 4-min FST session was measured 30 min after administration (n = 7–8).

Next, we examined structure-activity relationship of antidepressant activity of YL. Leu-Tyr (LY), a retrosequence dipeptide, and tyrosine and leucine, by themselves were ineffective, suggesting that the amino acid sequence is important for the antidepressant-like effect (F(4, 32) = 4.783, *p* < 0.05, Fig. 7a). We found previously that other aromatic amino acid-leucine dipeptides also exhibited anxiolytic activity. We examined depression-like activity of the peptides, Phe-Leu (FL) and Trp-Leu (WL). FL and WL were found to significantly decrease immobility time in the FST (F(3, 29) = 5.786, *p* < 0.05, Fig. 7b). On the other hand, Leu-Phe (LF) and Leu-Trp (LW) had no effect on immobility time (data not shown). These results suggested that aromatic amino acid-leucine was involved in the antidepressant-like activity.

We investigated antidepressant-like effect of YLG, an bovine milk casein-derived tripeptide-including YL sequence reported as an anxiolytic peptide. We found that YLG administration decreased immobility time in the FST (F(4, 51) = 6.812, *p* < 0.05, Fig. 7c). The intensity of the activity of YLG was approximately the same as that of YL.

## Discussion

We found that a dipeptide, YL, exhibits a potent antidepressant-like effect after peripheral administration in behavioral tests, comparable to that of antidepressants, imipramine and fluvoxamine. In addition, centrally administered YL exhibited an antidepressant-like effect. Although endogenous peptide hormones, such as leptin, ghrelin, insulin, and peptide YY, cross the blood-brain barrier (BBB)^23^, it is not clear whether YL crosses the BBB; however, brain-transportable dipeptides have recently been reported^24^. Thus, it cannot be ruled out that YL reaches the brain in dipeptide-form and act on the central nervous system (CNS). Further investigations are needed to clarify whether YL crosses the BBB and the mechanism of its antidepressant-like effects.

We found that YL administration increased BrdU-positive cells in the hippocampus of mice. Additionally, YL increased BrdU-positive cells in cultured hippocampal neural stem cells. Thus, we confirmed that YL directly promoted the proliferation of hippocampal progenitor cells. It was reported that hippocampal neuronal proliferation observed in adults decreases in patients with depression, and that enhancement of the proliferation of hippocampal progenitor cells contributes to improvements seen after treatment with antidepressants^25–28^. These results suggest that YL mimics antidepressants to enhance neuronal proliferation.

BDNF is known to mediate the proliferation of hippocampal progenitor cells and a fast-acting antidepressant-like effect induced by ketamine, an antagonist of glutamate N-methyl-D-aspartate (NMDA) receptors^29,30^. It was also reported that the BDNF-mediated antidepressant-like effect was independent of the HPA axis^31^, which is activated by stress.

In contrast, YL, which acts independently of BDNF, suppressed the stress-induced activation of the HPA axis. As HPA axis activation is also reported to suppress the proliferation of hippocampal progenitor cells^31–35^, the possibility that YL-induced suppression of HPA axis activation in response to stress contributes to the proliferation of hippocampal progenitor cells *in vivo* cannot be ruled out. In addition, our study found neuronal proliferation after acute administration of YL, but further studies are necessary to clarify whether YL induces neuronal differentiation and maturation after acute and chronic treatments, and whether YL modulates the BDNF system following long-term administration and/or in depression model mice.

The antidepressant-like effect of YL was blocked by antagonists of 5-HT_1A_, dopamine D_1_, and GABA_A_ receptors. It was reported that 5-HT_1A_ and dopamine D_1_ receptors in the hippocampus are involved in the antidepressant-like effect and hippocampal cell proliferation^28,36,37^. In the future, it will be necessary to elucidate how the monoamine system is directly and/or indirectly involved in the stress response and hippocampal cell proliferation.

A number of bioactive peptides have been isolated from digests of various food proteins. We previously predicted that there are anxiolytic-like peptides released by proteases present in the gastrointestinal tract, based on structure-activity relationship information^1,8,38^. We found that YLG was released after digestion by gastrointestinal proteases mimicking physiological conditions, and produce a potent anxiolytic-like effect via the activation of 5-HT_1A_, dopamine D_1_ and GABA_A_ receptors^8^. In addition, YLG improved a decline in cognition and a reduction in neural stem cell proliferation in mice on a high-fat diet^39^. These findings raise the possibility that bioactive peptides, including YLG, derived from food proteins potentially affect emotional behaviors via the activation of 5-HT_1A_, dopamine D_1_ and GABA_A_ receptors after a meal.

Similarly to YL, other aromatic amino acid-leucine dipeptides, FL and WL, also had antidepressant-like effects. However, the retrosequence peptide was ineffective. Thus, the aromatic amino acid-leucine sequence is important for the antidepressant-like effect. This structure-activity relationship of dipeptides in antidepressant-like activity is similar to that in anxiolytic-like activity, which we previously reported^1,38^. Furthermore, tripeptides with a YL sequence at the N-terminus, including YLQ and YLY, exhibited antidepressant-like effects, whereas LYL was ineffective (data not shown), suggesting that the YL sequence at the N-terminus is necessary for the potent antidepressant-like effects, and that C-terminal elongation of YL may not inhibit an antidepressant-like effect.

In summary, the dipeptide YL and the tripeptide YLG exhibited antidepressant-like effects in behavioral tests in mice after central and peripheral administration. These activities were orally active. YL promoted the proliferation of hippocampal progenitor cells independently of the BDNF pathway, and inhibited stress responses in the HPA axis.

## Methods

### Animals

Male ddY mice (24–27 g; SLC, Shizuoka, Japan) were reared in plastic cages in a room controlled with a 12-hour light-dark cycle (dark phase: 19:00–7:00), constant temperature (23 ± 1 °C), and constant humidity (55 ± 5%). The mice were housed for more than 3 days for acclimatization to the environment. They were fed regular tap water and a commercial solid diet (MF; Oriental Yeast, Osaka, Japan) ad libitum. All animal experiments were performed in accordance with the guideline of Kyoto University Ethics Committee for Animal Research Use. All animals were euthanized by an overdose of anesthesic or cervical spine dislocation after the experiment. This study was approved by Kyoto University Ethics Committee for Animal Research Use (approval number, 26–53).

### Reagents

Tyr-Leu (YL), Phe-Leu (FL), Trp-Leu (WL), Leu-Tyr (LY), Leu-Phe (LF), and Leu-Trp (LW) were purchased from Bachem AG (Bufendorf, Switzerland). Tyr-Leu-Gly (YLG) was synthesized by the Fmoc strategy, and purified by reverse-phase HPLC. Amino acids Tyr, Leu, Phe, and Trp were obtained from Wako Pure Chemical Industries, Ltd. (Osaka, Japan). WAY100135 dihydrochloride, a serotonin 5-HT_1A_ receptor antagonist; R(+)-SCH-23390 hydrochloride, a dopamine D_1_ receptor antagonist; and (−)-bicuculline methochloride and Flumazenil, γ-aminobutyric acid (GABA)_A_ receptor antagonists, were purchased from Tocris Bioscience (Bristol, UK).

### Forced swim test

The forced swim test (FST) was performed as previously described^40^. The total immobility time of mice was assessed according to Porsolt *et al*.^41^. Briefly, mice were individually forced to swim in an open cylindrical container (diameter 10 cm, height 20 cm), containing 10 cm of water at 25 ± 1 °C. The forced swim test was performed during the light phase of the light/dark cycle. The total immobility time (s) was measured using the SUPERMEX system (Muromachi Kikai Co., Ltd., Tokyo, Japan) in a time span of 2–6 min. Mice were considered immobile when they made no attempts to escape by movements to keep their heads above the water. A decrease in the immobility time was set as an indicator of an antidepressant-like effect. Previous studies showed that dipeptide and tripeptide affected brain functions, producing depressive, anxiety, and cognitive effects 30 min after peptide administration^1,8,38–40,42^. FST was started 30 min after administration of the peptide or saline orally, intracerebroventricularly, or intraperitoneally. WAY100135 dihydrochloride (10 mg/kg), R(+)-SCH-23390 hydrochloride (30 μg/kg), (−)-bicuculline methochloride (5 mg/kg), or Flumazenil (1 mg/kg) was coadministered i.p. once with the peptide 30 min prior to the behavioral test. Peptides and antagonists were dissolved in saline.

### Tail suspension test

Tail suspension test (TST) was performed as previously described^39^. The total duration of immobility induced by tail suspension was measured according to the method described by Steru *et al*.^43^. Briefly, mice were suspended 30 cm above the floor using adhesive tape placed approximately 1 cm from the tip of the tail. Immobility time was recorded during 2 to 6 min periods.

### c-Fos labeling

Synthesis of the c-Fos protein required 30 min of neuronal activity^44^. Control mice were administered the peptide sample or vehicle. Mouse brains were removed 1 h later. For immunohistochemistry, brains were fixed with 4% PFA and then immunostained with anti-c-Fos (1:1000; rabbit-IgG; Merck Millipore, Bedford, MA, USA) and biotin conjugated anti-rabbit IgG (1:1000, Jackson ImmunoReseach Inc., West Grove, PA, USA) antibodies. C-Fos-labeled cells were counted in the hippocampus under a microscope (Olympus, Tokyo, Japan).

### Hippocampal cell proliferation assay with BrdU *in vivo*

BrdU (Sigma, St. Louis, MO, USA) and peptide samples were dissolved in saline. Mice received i.p. administration of the peptide sample or vehicle 30 min before BrdU administration. Then, mice were administered BrdU (100 mg/kg; i.p.) to label proliferating cells in the hippocampus, and brains were removed 24 h later. For immunohistochemistry, brains were fixed with 4% PFA and then immunostained with anti-nestin (1:200; mouse-IgG; Chemicon, Temecula, CA, USA) and anti-BrdU (1:200; rat-IgG; Abcam Inc., Cambridge, MA, USA) antibodies, and DAPI. Alexa 488 conjugated anti-mouse and alexa 594 conjugated anti-rat (1:1000; Thermo Fisher Scientific Inc., San Jose, CA, USA) were used as secondary antibodies. BrdU-labeled cells were counted in the hippocampus under a microscope (Olympus).

### Hippocampal neural maturation assay with Dcx *in vivo*

Mice received i.p. administration of YL or vehicle, then brains were removed 24 hours later. For immunohistochemistry, brains were fixed with 4% PFA and then immunostained with an anti-doublecortin (Dcx) (1:1000; rabbit-IgG; Abcam) antibody. Alexa 488 conjugated anti-rabbit (1:1000; Thermo Fisher Scientific Inc.) was used as the secondary antibody. Dcx immunostaining intensity was measured using Image J software.

### Neuronal proliferation assay with BrdU *in vitro*

Neural progenitor cells were isolated from ddY mice at P1. The hippocampus was dissected free of the meninges and enzymatically digested with 0.25% trypsin-EDTA (Invitrogen) for 5 min at 37 °C, followed by washing with a 0.1% w/v trypsin inhibitor, aprotinin (Sigma). A single-cell suspension was plated in a 12-well culture plate. The culture medium was composed of DMEM/Ham’s F12 (1:1; Sigma), B27 supplement (Invitrogen), GlutaMAX supplement (Invitrogen), penicillin-streptomycin solution (Invitrogen), 10 ng/ml basic fibroblast growth factor (bFGF; Pepro Tech, Rocky Hill, NJ, USA), and 20 ng/ml epidermal growth factor (EGF; Pepro Tech). Neural progenitor cells were grown as free-floating neurospheres for 7 days in a 5% CO_2_, 95% air humidified atmosphere. Neurospheres were collected and then digested with 0.25% trypsin-EDTA for 5 min at 37 °C, followed by washing with 0.1% w/v trypsin inhibitor. Digested cells were plated on poly-l-ornithine- and fibronectin-coated 8-well culture dishes in serum-free neurobasal (NB) medium (Invitrogen) containing B27 supplement, GlutaMAX supplement, penicillin-streptomycin solution, 10 ng/ml bFGF, and 20 ng/ml EGF, and cultured for 3 days until used in the neuronal proliferation assay with BrdU.

Peptide or PBS was added in NB medium containing B27 supplement. After the addition of the peptide, cells were incubated in NB medium containing B27 supplement and 20 μM BrdU for 24 h. For immunocytochemistry, cultured cells were fixed with 4% PFA and then immunostained with anti-nestin (1:200; mouse-IgG; Chemicon) and anti-BrdU (1:200; rat-IgG; Abcam) antibodies, and DAPI. Alexa 488 conjugated anti-mouse and alexa 594 conjugated anti-rat (1:1000; Thermo Fisher Scientific Inc., San Jose, CA, USA) were used as secondary antibodies.

### Real-time RT-PCR

Hippocampal BDNF and hypothalamic CRH mRNA expression levels were measured using real-time RT-PCR. Control mouse hippocampus and hypothalamus was excised 30 min after i.p. administration of YL and was kept in RNAlater RNA Stabilization Reagent (QIAGEN Sciences Inc., Germantown, MD, USA) after decapitation under deep anesthesia until RNA extraction. Total RNA was extracted from the hippocampus using the RNeasy Lipid Tissue Kit (QIAGEN Sciences Inc.) and transcribed to cDNA with random primers using Takara PrimeScript® RT Master Mix (Takara, Osaka, Japan). For quantitative PCR, we amplified the cDNA using the Prism 7000 Sequence Detection System (Applied Biosystems, Foster City, CA, USA) with Platinum SYBR Green qPCR SuperMix-UDG and ROX solution (Invitrogen) and each primer set specific for mouse BDNF and CRH, according to the manufacturer’s instructions. The following primers were used for real-time RT-PCR: forward *bdnf*: 5′-GCG GCA GAT AAA AAG ACT GC-3′, reverse *bdnf*: 5′-TCA GTT GGC CTT TGG ATA CC-3′, forward *crh*: 5′-GCA GTT AGC TCA GCA AGC TCA C-3′, reverse *crh*: 5′-CAA ATG ATA TCG GAG CTG CG-3′, forward *β-actin*: CTG CGC AAG TTA GGT TTT GTC A, reverse *β-actin*: TGC TTC TAG GCG GAC TCT TAC TG. The reactions were cycled 40 times with denaturation at 95 °C for 15 s, and with annealing and elongation at 60 °C for 30 s. The relative expression level of each mRNA was normalized against the mRNA expression level of β-actin.

### ELISA

Control mouse hippocampus was excised 15, 30, 60, 120 min, 24 h after i.p. administration of YL. Blood was collected 30 min after i.p. administration of YL. Hippocampal BDNF protein and plasma corticosterone levels were measured by ELISA. The hippocampus was recovered and homogenized with ice-cold NP40 cell lysis buffer (Invitrogen). Blood was collected in a tube rinsed with heparin under deep anesthesia, then centrifuged (1000 g × 10 min at 4 °C). BDNF levels of the protein extract were measured using the Promega BDNF Emax® ImmunoAssay System (Promega, Madison, WI, USA) according to the manufacturer’s instructions. Corticosterone levels in the plasma were measured using the Assaypro corticosterone ELISA kit (Assaypro Llc, Brooklyn, NY, USA) according to the manufacturer’s instructions.

### Statistical analysis

All values are expressed as the means ± S.E.M. One-way analysis of variance (ANOVA) followed by Tukey-Kramer’s test was used to assess differences among three or more groups. Unpaired *t*-tests were used to assess differences between two groups. P values less than 0.05 were considered significant.

## References

[1] Kanegawa N, Suzuki C, Ohinata K (2010). Dipeptide Tyr-Leu (YL) exhibits anxiolytic-like activity after oral administration via activating serotonin 5-HT_1A_, dopamine D_1_ and GABA_A_ receptors in mice. FEBS Lett..

[2] Hirata H (2007). Rubiscolin-6, a δ opioid peptide derived from spinach Rubisco, has anxiolytic effect via activating σ_1_ and dopamine D1 receptors. Peptides.

[3] Ohinata K, Agui S, Yoshikawa M (2007). Soymorphins, novel μ opioid peptides derived from soy β-conglycinin β-subunit, have anxiolytic activities. Biosci. Biotechnol. Biochem..

[4] Zhao H, Ohinata K, Yoshikawa M (2008). Rubimetide (Met-Arg-Trp) derived from Rubisco exhibits anxiolytic-like activity via the DP_1_ receptor in male ddY mice. Peptides.

[5] Hou IC (2011). β-Lactotensin derived frombovine β-lactoglobulin exhibits anxiolytic-like activity as an agonist for neurotensin NTS2 receptor via activation of dopamine D1 receptor in mice. J. Neurochem..

[6] Mori Y (2018). Characterization of soy-deprestatin, a novel orally active decapeptide that exerts antidepressant-like effects via gut-brain communication. FASEB J..

[7] Mizushige T (2019). Ginger-Degraded Collagen Hydrolysate Exhibits Antidepressant Activity in Mice. J. Nutr. Sci. Vitaminol..

[8] Tanti A, Belzung C (2010). Open questions in current models of antidepressant action. Br. J. Pharmacol..

[9] Eisch AJ, Petrik D (2012). Depression and hippocampal neurogenesis: a road to remission?. Sci..

[10] Sahay A, Hen R (2007). Adult hippocampal neurogenesis in depression. Nat. Neurosci..

[11] Hanson ND, Owens MJ, Nemeroff CB (2011). Depression, antidepressants, and neurogenesis: a critical reappraisal. Neuropsychopharmacology.

[12] Tzeng WY (2013). Companions reverse stressor-induced decreases in neurogenesis and cocaine conditioning possibly by restoring BDNF and NGF levels in dentate gyrus. Psychoneuroendocrinology.

[13] Wang L, Burger LL, Greenwald-Yarnell ML, Myers MG, Moenter SM (2018). Glutamatergic Transmission to Hypothalamic Kisspeptin Neurons Is Differentially Regulated by Estradiol through Estrogen Receptor α in Adult Female Mice. J. Neurosci..

[14] Eerola K (2018). Hypothalamic γ-melanocyte stimulating hormone gene delivery reduces fat mass in male mice. J. Endocrinol..

[15] van den Pol AN, Acuna C, Davis JN, Huang H, Zhang X (2019). Defining the caudal hypothalamic arcuate nucleus with a focus on anorexic excitatory neurons. J. Physiol..

[16] Shirayama Y (2002). Brain-derived neurotrophic factor produces antidepressant effects in behavioral models of depression. J. Neurosci..

[17] Monteggia LM (2004). Essential role of brain-derived neurotrophic factor in adult hippocampal function. Proc. Natl Acad. Sci. USA.

[18] Li Y (2008). TrkB Regulates Hippocampal Neurogenesis and Governs Sensitivity to Antidepressive Treatment. Neuron.

[19] Schloesser RJ, Manji HK, Martinowich K (2009). Suppression of Adult Neurogenesis Leads to an Increased HPA Axis Response. Neuroreport.

[20] Butterweck V, Winterhoff H, Herkenham M (2001). St. John’s wort, hypericin, and imipramine: a comparative analysis of mRNA levels in brain areas involved in HPA axis control following short-term and long-term administration in normal and stressed rats. Mol. Psychiatry.

[21] Moncek F, Duncko R, Jezova D (2003). Repeated citalopram treatment but not stress exposure attenuates hypothalamic-pituitary-adrenocortical axis response to acute citalopram injection. Life Sci..

[22] Mizushige T, Sawashi Y, Yamada A, Kanamoto R, Ohinata K (2013). Characterization of Tyr-Leu-Gly, a novel anxiolytic-like peptide released from bovine αS-casein. FASEB J..

[23] Morita-Takemura S, Wanaka A (2019). Blood-to-brain communication in the hypothalamus for energy intake regulation. Neurochem. Int..

[24] Tanaka M (2019). Brain-transportable dipeptides across the blood-brain barrier in mice. Sci. Rep..

[25] Campbell S, Macqueen G (2004). The role of the hippocampus in the pathophysiology of major depression. J. Psychiatry Neurosci..

[26] Czéh B, Lucassen PJ (2007). What causes the hippocampal volume decrease in depression? Are neurogenesis, glial changes and apoptosis implicated?. Eur. Arch. Psychiatry Clin. Neurosci..

[27] Lucassen PJ (2010). Decreased numbers of progenitor cells but no response to antidepressant drugs in the hippocampus of elderly depressed patients. Neuropharmacol..

[28] Park. SC (2019). Neurogenesis and antidepressant action. Cell Tissue Res..

[29] Castrén E, Võikar V, Rantamäki T (2007). Role of neurotrophic factors in depression. Curr. Opin. Pharmacol..

[30] Autry AE (2011). NMDA receptor blockade at rest triggers rapid behavioural antidepressant responses. Nat..

[31] Hashimoto K, Shimizu E, Iyo M (2004). Critical role of brain-derived neurotrophic factor in mood disorders. Brain Res. Brain Res Rev..

[32] Brown ES, Rush AJ, McEwen BS (1999). Hippocampal remodeling and damage by corticosteroids: implications for mood disorders. Neuropsychopharmacology.

[33] Snyder JS (2011). Adult hippocampal neurogenesis buffers stress responses and depressive behaviour. Nat..

[34] Danzer SC (2012). Depression, stress, epilepsy and adult neurogenesis. Exp. Neurol..

[35] Schoenfeld TJ, Gould E (2012). Stress, stress hormones, and adult neurogenesis. Exp. Neurol..

[36] Mineur YS (2015). Expression of the 5-HT1A Serotonin Receptor in the Hippocampus Is Required for Social Stress Resilience and the Antidepressant-Like Effects Induced by the Nicotinic Partial Agonist Cytisine. Neuropsychopharmacology.

[37] Mishra A, Singh S, Tiwari V, Parul, Shukla S (2019). Dopamine D1 receptor activation improves adult hippocampal neurogenesis and exerts anxiolytic and antidepressant-like effect via activation of Wnt/β-catenin pathways in rat model of Parkinson’s disease. Neurochem. Int..

[38] Mizushige T (2013). Aromatic amino acid-leucine dipeptides exhibit anxiolytic-like activity in young mice. Neurosci. Lett..

[39] Nagai, A., Mizushige, T., Matsumura, S., Inoue, K. & Ohinata, K. Orally administered milk-derived tripeptide improved cognitive decline in mice on a high-fat diet. *FASEB J*, In press. (2019). 10.1096/fj.201900621R 31652095

[40] Yamamoto Y (2015). Antidepressant-like effect of food-derived pyroglutamyl peptides in mice. Neuropept..

[41] Porsolt RD, Le Pichon M, Jalfre M (1977). Depression: a new animal model sensitive to antidepressant treatments. Nat..

[42] Yamada A, Mizushige T, Kanamoto R, Ohinata K (2014). Identification of novel β-lactoglobulin-derived peptides, wheylin-1 and -2, having anxiolytic-like activity in mice. Mol. Nutr. Food Res..

[43] Steru L, Chermat R, Thierry B, Simon P (1985). The tail suspension test: a new method for screening antidepressants in mice. Psychopharmacol..

[44] Abbadie C, Taylor BK, Peterson MA, Basbaum AI (1997). Differential contribution of the two phases of the formalin test to the pattern of c-fos expression in the rat spinal cord: studies with remifentanil and lidocaine. Pain..

